# Helminth Genomics: The Implications for Human Health

**DOI:** 10.1371/journal.pntd.0000538

**Published:** 2009-10-26

**Authors:** Paul J. Brindley, Makedonka Mitreva, Elodie Ghedin, Sara Lustigman

**Affiliations:** 1 Department of Microbiology, Immunology, and Tropical Medicine, George Washington University Medical Center, Washington, D. C., United States of America; 2 Genome Centre and Department of Genetics, Washington University School of Medicine, St. Louis, Missouri, United States of America; 3 Division of Infectious Diseases, University of Pittsburgh School of Medicine, Pittsburgh, Pennsylvania, United States of America; 4 New York Blood Center, Laboratory of Molecular Parasitology, New York, New York, United States of America; Biomedical Research Institute, United States of America

## Abstract

More than two billion people (one-third of humanity) are infected with parasitic roundworms or flatworms, collectively known as helminth parasites. These infections cause diseases that are responsible for enormous levels of morbidity and mortality, delays in the physical development of children, loss of productivity among the workforce, and maintenance of poverty. Genomes of the major helminth species that affect humans, and many others of agricultural and veterinary significance, are now the subject of intensive genome sequencing and annotation. Draft genome sequences of the filarial worm *Brugia malayi* and two of the human schistosomes, *Schistosoma japonicum* and *S. mansoni*, are now available, among others. These genome data will provide the basis for a comprehensive understanding of the molecular mechanisms involved in helminth nutrition and metabolism, host-dependent development and maturation, immune evasion, and evolution. They are likely also to predict new potential vaccine candidates and drug targets. In this review, we present an overview of these efforts and emphasize the potential impact and importance of these new findings.

## Helminth Infections—The Great Neglected Tropical Diseases

**Figure 1:**
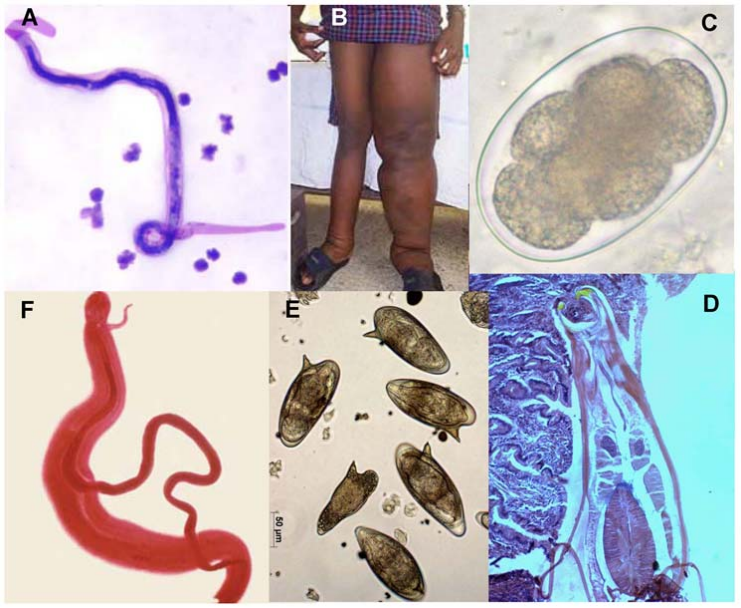
Montage of some of the major human helminth parasites, their developmental stages, and disease pathology. (A) Microfilaria of *Brugia malayi* in a thick blood smear, stained with Giemsa (http://www.dpd.cdc.gov/dpdx/html/frames/a-f/filariasis/body_Filariasis_mic1.htm); the microfilaria is about 250 µm in length. (B) Patient with lymphedema of the left leg due to lymphatic filariasis (http://www.cdc.gov/ncidod/dpd/parasites/lymphaticfilariasis/index.htm). (C) Hookworm egg passed in the stool of an infected person; the microscopic egg, barrel-shaped with a thin wall, is about 70×40 µm in dimension. (D) longitudinal section through an adult hookworm attached to wall of small intestine, ingesting host blood and mucosal wall. The parasite is about 1 cm in length. (E) Eggs of *Schistosoma mansoni*. The egg is about 150×50 µm in dimension; the lateral spine is diagnostic for *S. mansoni* in comparison to the other human schistosome species. Fibrotic responses to schistosome eggs trapped in the intestines, liver, and other organs of the infected person are the cause of the schistosomiasis pathology and morbidity. (F) A pair of adult worms of the blood fluke *Schistosoma mansoni*; the more slender female worm resides in the gynecophoral canal of the thicker male. The worms are about 1.5 cm in length, and live for many years (http://www.dpd.cdc.gov/dpdx/HTML/ImageLibrary/Schistosomiasis_il.htm ).

**Figure 2:**
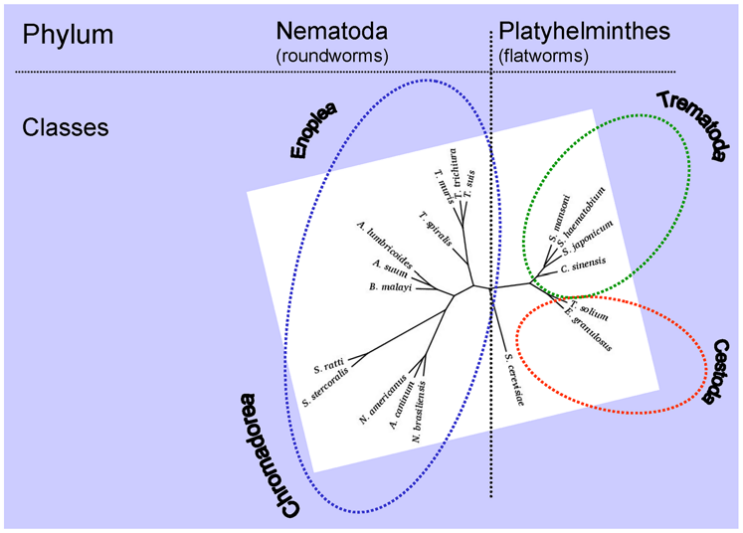
Phylogeny of the major taxa of human helminths—nematodes and platyhelminths—as established by maximum likelihood (ML) analysis of 18S ribosomal RNA from 18 helminth species. Sequences were aligned using ClustalX [93]. The topology of the tree was derived from a consensus tree by neighbor-joining–based bootstrapping, its branch lengths were computed using a ML-based method, and it was rooted with the orthologue from the brewer's yeast, *Saccharomyces cerevisiae*. The branch lengths of the phylogenetic tree were computed using DNAML (PHYLIP package [94]) by allowing rate variation among sites. The headings Chromadorea, Enoplea, Trematoda, and Cestoda are major classes of the phyla Nematoda and Platyhelminthes. The GenBank accession numbers of aligned sequences are DQ118536.1 (*Trichuris trichiura*), AY851265.1 (*Trichuris suis*), AF036637.1 (*Trichuris muris*), AY497012.1 (*Trichinella spiralis*), U94366.1 (*Ascaris lumbricoides*), AF036587.1 (*Ascaris suum*), AF036588.1 (*Brugia malayi*), AJ920348.1 (*Necator americanus*), AJ920347.2 (*Ancylostoma caninum*), AF036597.1 (*Nippostrongylus brasiliensis*), X03680.1 (*Caenorhabditis elegans*), AF036605.1 (*Strongyloides ratti*), U81581.1 (*Strongyloides ratti*), AB453329.1 (*Strongyloides ratti*), AF279916.2 (*Strongyloides stercoralis*), AB453315.1 (*Strongyloides stercoralis*), M84229.1 (*Strongyloides stercoralis*), EU011664.1 (*Saccharomyces cerevisiae*), , U27015.1 (*Saccharomyces cerevisiae*), DQ157224.1 (*Taenia solium*), AF229852.1 (*Clonorchis sinensis*), Z11590.1 (*Schistosoma japonicum*), Z11976.1 (*Schistosoma haematobium*), U65657.1 (*Schistosoma mansoni*).

Helminth parasites are parasitic worms from the phyla Nematoda (roundworms) and Platyhelminthes (flatworms) (Figures 1 and 2); together, they comprise the most common infectious agents of humans in developing countries. The collective burden of the common helminth diseases—which range from the dramatic sequelae of elephantiasis and blindness to the more subtle but widespread effects on child development, pregnancy, and productivity—rivals that of the main high-mortality conditions such as HIV/AIDS or malaria [1]. For example, based on a recent analysis [2], 85% of the neglected tropical disease (NTD) burden for the poorest 500 million people living in sub-Saharan Africa (SSA) results from helminth infections. Hookworm infection occurs in almost half of the poorest people in SSA, including 40–50 million school-aged children and 7 million pregnant women, in whom it is a leading cause of anemia. Schistosomiasis (192 million cases) is the second most prevalent NTD after hookworm, accounting for 93% of the world's number of cases of schistosomiasis and possibly associated with increased horizontal transmission of HIV/AIDS. Lymphatic filariasis (46–51 million cases) and onchocerciasis (37 million cases) are also widespread in SSA, each disease representing a significant cause of disability and reduction in the region's agricultural productivity. The disease burden estimate in disability-adjusted life years (DALYs) for total helminth infections in SSA is 5.4–18.3 million in comparison to 40.9 million DALYs for malaria and 9.3 million DALYs for tuberculosis. Yet, research into helminth infections has not received nearly the same level of support. This is partly because helminthiases are diseases of the poorest people in the poorest regions, but also because these pathogens are difficult to study in the laboratory by comparison to most model eukaryotes and many other pathogens. Standard tools and approaches, including cell lines, culture in vitro, and animal models, are generally lacking. In addition, the genomes of helminths are generally much more complex than those of model organisms like yeast and fruit flies [2].

Whereas helminth diseases are ancient scourges of humanity, with some known from biblical times, most can also be considered as re-emerging diseases in the sense that new outbreaks are reported routinely in response to environmental and sociopolitical changes [3]. For example, schistosomiasis has reemerged many times in Africa in recent times in response to hydrological changes, e.g. construction of dams, irrigation canals, reservoirs, etc. that establish suitable new environments for the intermediate host snails that transmit the parasites. Schistosomiasis has also reemerged in mountainous and hilly regions in Sichuan, China, where it had been controlled previously by intensive interventions [4]. Furthermore, new strains of schistosomes are indeed emerging through natural hybridizations between human and cattle species of schistosomes [5].

**Figure 3:**
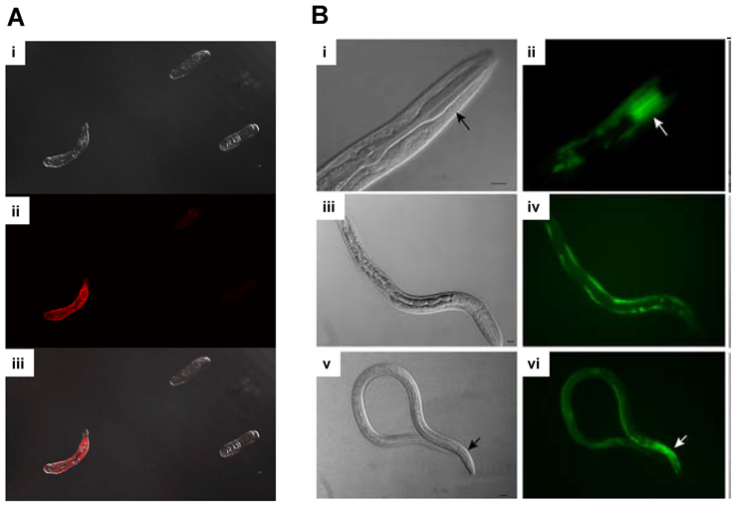
Some recent approaches to expressing transgenes in human helminths. (A) Luciferase activity in *Schistosoma mansoni* larvae (schistosomules) after transduction with a pseudotyped retrovirus that expresses the luciferase reporter gene. Anti-luciferase antibody staining of schistosomules three days after exposure to pseudotyped lentivirus carrying the firefly luciferase transgene. Schistosomules examined by confocal laser microscopy; (i) bright field, (ii) fluorescence red channel, (iii) merged images. Control non-transformed worms showed only background levels of fluorescence (not shown; see [34]–[36] for relevant hypotheses and experimental methods). (B) Recent studies on transgenic *Strongyloides stercoralis* indicated that morphogenesis of the infective L3 stage larva requires the DAF-16 orthologue FKTF-1 [38]. L3s of this parasitic nematode were transfected with plasmids carrying the transgene fktf-1*b*::gfp::fktf-1b and examined by fluorescence microscopy. (i, ii) Transgenic first-stage larvae express green fluorescent protein (GFP) in the procorpus (arrow) of the pharynx. (iii, iv) A first-stage larva (L1) expresses the GFP::FKTF-1b(wt) transgene in the hypodermis. (v, vi) An infective L3 expresses the GFP::FKTF-1b(wt) fusion protein in the hypodermis and in a narrow band in the pharynx (arrow). Scale bars, 10 µm. Adapted from [38].

Despite the difficulties with investigation of helminth parasites, new insights into fundamental helminth biology are accumulating through genome projects and the application of genome manipulation technologies including RNA interference and transgenesis (Figure 3). What's more, research on immunology of helminth infections has contributed enormously to our understanding of Th2 immune responses, the function of regulatory T cells, generation of alternatively activated macrophages, and the transmission dynamics of infectious agents. It is hoped that this progress can be translated into new and robust drugs, diagnostics, and vaccines for the helminth diseases of humanity and those of our livestock and companion species [1], [6]–[10].

## Genomics Approaches to Investigating Helminths

Over the past decade, increasing numbers of helminth-specific genome sequences have become available due to ever-improving techniques for obtaining biological material, extracting RNA and DNA, constructing complimentary DNA (cDNA)/whole genome shotgun libraries, and, especially, major advances in the chemistry and instrumentation for DNA sequencing and its concomitant decreased cost. Helminth genomics began with the generation and analysis of transcribed sequences (expressed sequence tags [ESTs] [11]), which has proved to be a rapid and cost-effective route to discover genes in other eukaryotes. In April 2009, there were ∼550,000 nematode and 450,000 platyhelminth ESTs in the dbEST division of GenBank, excluding those from the model nematode *Caenorhabditis rhabditis*. Of these, 60% were from parasites of humans and closely related animal pathogens used to study human infections (Table 1). These ESTs have many applications. They can be used to annotate helminth genomes (see below) to determine alternative splicing, verify open reading frames, and confirm exon/intron and gene boundaries. They are valuable also, for example, in functional genomics to design probes for expression microarrays (e.g., [12]) and to provide putative protein sequence information for proteomics methods (e.g., [13]), to name but a few applications. Quantitative analysis of ESTs (transcriptomics), including serial expression of gene analysis, can identify transcripts that are either over- or under-represented by comparison to other transcripts in various helminth life cycle stages or tissues (e.g., [14],[15]), and the subset of genes evaluated with gene ontology programs provide insights into cellular and metabolic pathway functioning in the parasite (e.g., [16]). Furthermore, one can identify potential targets for interventions by applying a hierarchy of considerations including a matrix of biological, expression, and phenotypic data [17] or by performing a pan-phylum analysis to identify conserved parasite-specific genes whose selective targeting will have low or no toxicity to the host [18],[19] or genes that have diverged enough from the host counterpart, resulting in altered or absent functions [20].

**Table 1:** Human parasitic helminths (and their close relatives) with genome sequencing projects completed or underway.

Phylum or Class	Species	Common Name / Disease	Primary host	Genome size, Mb	GenBank Project ID	cDNAs (3730 ABI), 1,000 s	Genome Sequencing Status	Sequencing Institute^a^
**Nematoda (roundworms)**								
Clade V^b^	*Necator americanus*	Hookworm/necatoriasis	Human	—	20369	5	In progress	WUGC
	*Ancylostoma caninum*	Model hookworm	Dog	344	12841	81	Improving draft	WUGC
	*Nippostrongylus brasiliensis*	Model hookworm	Rat	—	20445	14.7	In progress	SI
Clade IV	*Strongyloides stercoralis*	Threadworm/strongyloidiasis	Human	—	—	11.4	In progress	SI
	*S. ratti*	Model threadworm	Rat	—	—	27.4	In progress	SI/WUGC
Clade III	*Ascaris lumbricoides*	Large roundworm/ascariasis	Human	230	—	1.8	In progress	SI
	*A. sum*	Model large roundworm	Pig	230	—	55.7	Improving draft	WUGC/SI
	*Brugia malayi*	Filaria/lymphatic filariasis	Human	96	9549	26.2	Improving draft	TIGR/University of Pittsburgh
	*Loa Loa*	Filaria/loaisis (cutaneous filariasis)/African eye worm	Human	—	—	3.3	In progress	BI
	*Onchocerca volvulus*	Filaria/river blindness	Human	150	—	15	In progress	SI
	*Acanthocheilonema viteae*	Model filaria	Rodent	—	33239	0	In progress	UMIGS
Clade I	*Trichinella spiralis*	Trichina worm/trichinosis	Pig to human	71	12605	25.3	Draft completed	WUGC
	*Trichuris trichiura*	Whipworm/trichuriasis	Human	—	—	0	In progress	SI
	*T. muris*	Model whipworm	Mouse	96	—	7	In progress	SI
	*T. suis*	Model whipworm	Pig	-	—	0	In progress	WUGC
**Cestoda (tapeworms)**	*Echinococcus multilocularis*	Tapeworm/alveolar hydatidosis	Rodent; larva infects humans	150	—	1	In progress	SI
	*E. granulosus*	Tapeworm/unilocular hydatidosis	Canids; larva infects humans	150	12620	10	In progress	SI
	*Taenia solium*	Pork tapeworm/taeniasis/cysticercosis	Human	270	17815	25	Draft completed	Mexico City
**Trematoda (flukes)**	*Schistosoma mansoni*	Blood fluke/intestinal schistosomiasis	Human	390	12599	206	Draft completed	SI/TIGR
	*S. haematobium*	Blood fluke/urinary schistosomiasis	Human	—	12616	0	In progress	SI
	*S. japonicum*	Blood fluke/intestinal schistosomiasis	Human	400	29491	104	Draft completed	CNHGC
	*Clonorchis sinensis*	Liver fluke/clonorchiasis	Human	—	17975	3	In progress	SNUCM

a WUGC, Washington University's Genome Center.

b Phylogeny based on Blaxter et al. [47].

BI, Broad Institute; CNHGC, Chinese National HGC; SI, Sanger Institute; SNUCM, Seoul National University College of Medicine; TIGR, The Institute for Genomic Research (now JCVI).

The first multicellular genome sequenced was that of the free-living roundworm *C. elegans* [21]; reported in 1998, it is still the only metazoan for which the sequence of every nucleotide is known with high confidence. Today, the genome sequences of 22 species of helminths that either infect humans or are closely related parasites are completed or underway (Table 1). A comprehensive genome analysis has been published for several of them, including the lymphatic filarial nematode *Brugia malayi* [22], the dog hookworm *Ancylostoma caninum* [23], and the blood flukes *Schistosoma japonicum* and *S. mansoni* [24],[25] (Figure 1; Table 1). Some of the main obstacles to research on human parasites are their life cycle complexity, tissue complexity, and the paucity of genetic and transgenic methods for manipulating genes of interest. Comparative genome analyses have also provided insights into the adaptations of various parasites to niches in their human (and vector) hosts as well as insights into the molecular basis of the mutualistic relationship between the filarial nematode *B. malayi* and its endosymbiont *Wolbachia* (see below).

The genomes of the schistosomes *S. japonicum* and *S. mansoni* are the first complete genomes reported for members of the Lophotrochozoa [24],[25], a large taxon that includes about 50% of all metazoan phyla including the mollusks, annelids, brachiopods, nemerteans, bryozoans, playthelminths, and others [26]. These schistosome genome sequences revealed remarkable features of the host–parasite relationship. Among these, the schistosome genome has lost numerous protein-encoding domains. Whereas the total number (∼6,000) of protein families is broadly similar among schistosomes, humans, *C. elegans*, and fruit fly, about 1,000 protein domains have been abandoned by *S. japonicum*, including some involved in basic metabolic pathways and defense, implying that loss of these domains could be a consequence of the adoption of a parasitic way of life. If so, the remaining molecular repertoire must have evolved in parallel with this extensive domain loss to permit the pathogen to locate and infect humans efficiently, nourish itself, and interact with the external environment as well as with the host. On the other hand, despite extensive gene and domain loss, a number of schistosome gene families have expanded and these provide insights into the requirements for a parasitic lifestyle. Among the expanded gene families, a metalloprotease called invadolysin (or leishmanolysin) has at least 12 putative family members in schistosomes compared to a single orthologue in the human, fruit fly, and *C. elegans* genomes and only three in the free-living flatworm *S. mediterranea*. This protease family may facilitate skin penetration and tissue invasion by the cercaria, the infective-stage larva of the schistosome [24],[25].

Publication of genome sequences of filaria and schistosomes has underscored the pressing need to develop functional genomics approaches for these significant pathogens. Functional analyses—which use approaches such as RNA interference (RNAi) and translational studies—are essential to resolve uncertainties in the molecular physiology of helminths and to illuminate mechanisms of pathogenesis that may lead to development of new interventions to control and eliminate these parasites or the diseases. Progress in the functional genomics of helminths was reviewed recently [6],[27],[28]. In brief, RNAi has been used to inactivate the RNA products of several genes in schistosomes (e.g., [29]–[32]) and nematodes (e.g., [33]; reviewed in [8]). In addition, the recent genome sequences of *S. mansoni* and *S. japonicum* now make feasible genome-scale investigation of transgene integration into schistosome chromosomes. Gene therapy–like approaches to transform schistosomes include the use of the *piggyBac* transposon and pseudotyped murine leukemia retrovirus as transgene vectors [34]–[36] (Figure 3A), both of which offer a means to establish transgenic lines of schistosomes, to elucidate schistosome gene function and expression, and to advance functional genomics approaches for these parasites. Notably, progress is also being made to express reporter transgenes in parasitic nematodes including *Strongyloides stercoralis* [37], in which transgene approaches developed for use in *C. elegans* have recently been used to demonstrate that morphogenesis of infective larvae requires the DAF-16 orthologue FKTF-1 (Figure 3B) [38]. Progress is also being made with systems for analysis of promoter sequences of genes of parasitic nematodes (e.g., [39]).

Many future discoveries resulting from the parasitic helminths genome information can be expected to emanate from the broader scientific community rather than by the laboratory originating a genome sequence project. For the specialized genome sequence labs, dissemination of sequence information in a way that is useful, consistent, centralized, and lasting has been therefore a key goal. Efforts have gone well beyond depositing raw data in public databases. Currently, helminthologists have available a number of specialized sites for sequence analysis. *C. elegans* information is easily accessible at http://www.wormbase.org [40]. Useful information about the organism includes genome sequence, genetic and physical maps, transcript data (EST, mRNA, SAGE, TEC-RED, ORFeome, expression patterns from reporter gene fusions, and microarrays), the developmental lineage of all cells, connectivity of the nervous system, mutant phenotypes and genetic markers, gene expression described at the level of single cells, 3D protein structures, NCBI Clusters of Orthologous Groups, and apoptosis and aging information. It also contains extensive information from large-scale genomics analyses, including precomputed sequence similarity searches, protein motif analyses, protein–protein interactions, findings from systematic RNAi screens, single nucleotide polymorphisms (SNPs), orthologous and paralogous relationships, and the assignment of Gene Ontology (GO) terms to gene products. These resources greatly aid in the interpretation of much of the sequence data emerging from parasitic helminths.

However, accumulating evidence suggest that *C. elegans* is not a good model for all parasitic helminths, especially for the ones that are phylogenetically very distant such as the basic nematode and zoonotic parasite *Trichinella spiralis* (e.g., [41]). The other specialized site is Nematode.net (http://www.nematode.net) [42]), developed with a primary aim to disseminate the diverse collection of information for parasitic nematodes to the broader scientific community in a way that is useful, consistent, centralized, and enduring. In addition to sequence data, the site hosts assembled NemaGene clusters in GBrowse views, characterizing composition and protein homology, functional Gene Ontology annotations presented via the AmiGO browser, KEGG-based graphical display of NemaGene clusters mapped to metabolic pathways, codon usage tables, NemFam protein families (which represent conserved nematode-restricted coding sequences not found in public protein databases), and a Web-based WU-BLAST search tool that allows complex querying and other assorted resources. Furthermore, Nematode.net, by connecting data across the entire phylum Nematoda, has made a substantial contribution toward integrating the historically separate fields of *C. elegans*, vertebrate parasitology, and plant parasitology research. Finally, Nembase (http://www.nematodes.org [43]) also offers access to parasite sequence and tools such as visualization of clusters by stage of expression.

While each of these databases has been challenged by the requirement to support the influx of new genomes and related data, they nonetheless provide user communities with innovative features and tools suited to their needs that are beyond the scope of the large sequence repositories. For flatworms (Figure 2), it is notable that public genome annotation and analysis tools are already in place, including SchistoDB (http://schistoDB.net/), a genomic database for *S. mansoni* that incorporates sequences and annotation [44] and SjTPdb, http://function.chgc.sh.cn/sj-proteome/index.htm, an integrated transcriptome and proteome database and analysis platform for *S. japonicum* [45]. The genome database for the planarian *Schmidtea mediterranea*, a model free-living platyhelminth, can be expected to be advantageous to comparative genome projects and specific research problems for the growing number of parasitic flatworms that now are or will be subjects of genome sequence analysis. In addition, because of the phylogenetic position of planarians as early bilaterian metazoans, SmedGD (http://smedgd.neuro.utah.edu) will prove useful not only to planarian research, but also to investigations on developmental and evolutionary biology, comparative genomics (specifically with parasitic flatworms including flukes and tapeworms), stem cell research, and regeneration [46]).

## Evolution of Parasitism in Helminths

Genomics research has helped our understanding of the evolution of helminths of humans and other hosts, certainly with regard to roundworms of the phylum Nematoda. The first comprehensive study of the molecular evolution of helminths was a phylogenetic analysis of the small subunit ribosomal DNA (ss rDNA) sequences from 53 roundworms [47]. This study included both major parasitic and free-living taxonomic groups. It identified five major clades within the Nematoda and suggested that parasitism of animal and plants arose independently multiple times. A more recent study included 339 nearly full-length ss rDNAs and proposed subdivision of the phylum into 12 clades [48]. This revealed that nematodes that feed on fungi occupy a basal position compared to their plant parasite relatives, confirming that the parasitic nematodes of plants arose from fungivorous ancestors. Phylogenetic methods are also being used to study evolution of parasitism-related protein-coding genes (such as the enzymes that degrade the plant cell wall in nematode parasites of plants [cellulases, pectate lyases, etc.]) to understand better the mechanisms underlying the evolution of parasitism (reviewed in [49]). Recent genome-wide analysis of two plant parasitic nematodes [50],[51] provided a more complete picture of the acquisition of these cellulase genes, apparently by horizontal gene transfer (HGT) from prokaryotes. The subsequent expansion and diversification of HGT genes in these nematodes allow inferences about the evolutionary history of these parasites, and in addition present potential targets for anti-nematode drugs. When the genome of the necromenic nematode *Pristionchus pacificus* was reported recently, it became was clear that cellulases were not restricted to plant parasitic nematodes; their presence in this species indicated preadaptation for parasitism of animals [52], consistent with the intermediate evolutionary position of *Pristionchus* between the microbivorous *C. elegans* and the animal parasitic nematodes. In like fashion to evolution of parasitism among nematodes, we can predict that additional analyses of parasitic and free-living flatworm genomes will provide deeper insights into how and when parasitism evolved in the phylum Platyhelminthes, particularly in comparison to the fresh-water planarian *S. mediterranea*, a non-parasitic flatworm for which a draft genome is available [53]. In addition to evolution of parasitism of humans and other vertebrate hosts, helminth parasite genome sequences will also facilitate evolutionary studies on the role of intermediate hosts/vectors such as the snail in schistosome infections and the mosquito in filarial infections in this evolution.

## Host–Parasite Relationships

Investigations of regulatory networks involved in the embryonic development, organogenesis, development, and reproduction of helminths based on newly available genome sequences have revealed the presence of well-characterized signaling pathways, including those for Wnt, Notch, Hedgehog, and transforming growth factor β (TGF-β). These pathways can be recognized in the *B. malayi* and schistosome genomes [22],[24],[25]. These include endogenous hormones including epidermal growth factor (EGF)-like and fibroblast growth factors (FGF)-like peptides. Predicted components of the Ras–Raf–MAPK and TGF-β–SMAD signaling pathways (including FGF and EGF receptors), for example, encoded by these genomes, have components sharing high sequence identity with their mammalian orthologs, implying that schistosomes or filarial worms, in addition to utilizing their own pathways, might exploit host growth factors as developmental signals.

Immune regulation by helminth parasites includes suppression, diversion, and alteration of the host immune response, resulting in an anti-inflammatory environment that is favorable to parasite survival. For example, chronic infections induce key changes in host immune cell populations including dominance of the T-helper 2 (Th2) cells and selective loss of effector T cell activity, against a background of regulatory T cells, alternatively activated macrophages, and Th2-inducing dendritic cells [54],[55]. With advances in genomics, numerous parasite-derived proteins, including cytokine homologs, protease inhibitors, and an intriguing set of novel products, as well as glycoconjugates and small lipid moieties, have been discovered with known or hypothesized roles in immune interference [56]–[61]. These studies suggest that secreted parasite products interfere with different arms of the immune system by influencing the cytokine network and signal transduction pathways or by inhibiting essential enzymes. Using bioinformatics to compare the predicted proteome of *B. malayi* to proteins implicated in the immune response (interleukins, chemokines, and other signaling molecules), potential immune modulators produced by the filarial have been identified, including genes encoding the macrophage migration inhibition family of signaling proteins [62]. Furthermore, the genome of the blood fluke *S. mansoni* encodes a large array of paralogues of fucosyl and xylosyltransferases [25] that are involved in generating novel glycans at the host–parasite interface and could have an important role in the subverting the host immune system. A recent comprehensive review summarizes our current understanding of the growing number of individual helminth mediators that target key receptors or pathways in the mammalian immune system [63].

Helminth infection can have a broad impact on the entire immune system. Infection with trematode and nematode parasites, for example, correlates with a reduced incidence of atopic, allergic-type disorders [64]. Thus, helminth infection might potentially be useful as a novel therapy for allergic or autoimmune diseases [65]. Recently, worms, eggs, or purified nematode parasite protein have been used in preclinical and clinical trials to protect humans from allergy and autoimmunity (reviewed in [66]–[70]), including Crohn's disease and ulcerative colitis [71],[72]. Other studies have shown that substances produced by helminths, for example *Ascaris suum*, *Nippostrongylus brasiliensis,* and *Acanthocheilonema viteae*, can directly interfere with allergic responses or with development of allergen-specific Th2 responses [73]–[75]. ES-62, a molecule secreted by the filarial nematode *A. viteae*, directly inhibits the FceRI-induced release of mediators from mast cells, protects against mast-cell–dependent hypersensitivity in skin and lungs [76] and inhibits collagen-induced arthritis [77]. Research is underway to develop molecules that mimic the activity of ES-62 as drugs for allergic and autoimmune diseases [66]. Other helminth-derived products have the potential to reduce allergic responses. These products include schistosomal lysophosphatidylserine (lyso-PS) [61] and thioredoxin peroxidase from the liver fluke *Fasciola hepatica* [78]. These findings demonstrated that helminths produce products that can interfere with both the development of allergic responses and the workings of host effector mechanisms.

## The “Dependent” Helminth

As a consequence of evolution of an obligatory parasitic existence, helminth parasites are dependent upon their intermediate and definitive hosts for many necessities including nutrients such as amino acids; filariae are dependent on insect vectors to transport them to the host. The newly available genome sequences for schistosomes and *B. malayi* have confirmed earlier biochemical studies that had revealed aspects of physiological/ biochemical dependence of these parasites on the host. For example, schistosomes cannot synthesize fatty acids de novo, or sterols, purines, and nine human essential amino acids plus arginine or tyrosine, and must catabolize complex precursors obtained from their hosts. Loss or degeneracy of fatty acid, sterol, and purine synthesis pathways in schistosomes likely relates to the adoption of a parasitic lifestyle; it is notable that genes encoding all the key enzymes for both the de novo fatty acid and purine syntheses are complete in the (free-living) planarian *S. mediterranea*. To obtain essential lipid nutrients, the schistosome genome encodes transporters, including apolipoproteins, low-density lipoprotein receptor, scavenger receptor, fatty-acid-binding protein, ATP-binding-cassette transporters and cholesterol esterase, to exploit fatty acids and cholesterol from host blood [25],[79].

Many species of filarial nematodes are themselves infected by the endosymbiotic bacterium *Wolbachia*. The genome sequence of the *Wolbachia* species that infects the roundworm nematode *B. malayi* (wBm) [80] helped establish which metabolites the bacterium potentially provides to the nematode (riboflavin, flavine adenine dinucleotide, heme, and nucleotides, for example) and which are provided by the nematode to the endobacterium (notably, amino acids). This type of information has opened up the exciting possibility that drugs already registered for human use might inhibit key biochemical pathways in *Wolbachia* that could sterilize or kill the adult worms. Although the *Wolbachia* genome is even more degenerate than that of the related pathogen *Rickettsia*, it has retained more intact metabolic pathways than *Rickettsia*. This may be important in its biochemical contribution to host (i.e., filarial) viability and fecundity.

The wBm genome encodes many more proteases and peptidases than *Rickettsia*, which likely degrade host proteins in the extracellular environment. Other proteins encoded by wBm include a common type IV secretion system, as used by some pathogenic gram-negative bacteria to transfer plasmids and proteins into surrounding host cells, and an abundance of ankyrin domain-containing proteins, which might regulate host gene expression, as suggested for *Ehrlichia phagocytophilia* AnkA [81], as well as several proteins predicted to localize on the cell surface. Ankyrin domain–containing proteins are noteworthy because of their roles in protein–protein interactions in a variety of cellular processes. A number of other wBm molecules are of interest as potential drug targets. For example, glutathione biosynthesis genes may provide glutathione for the protection of the filaria from oxidative stress or human immunological effector molecules. Heme produced from wBm (all five synthesis genes are present) could be vital to worm embryogenesis, as there is evidence that molting and reproduction are controlled by ecdysteroid-like hormones [82], synthesis of which requires heme. Depletion of *Wolbachia* might therefore halt production of these hormones and block molting and/or embryogenesis in *B. malayi*. Most, if not all, nematodes, including *B. malayi*, appear to be unable to synthesize heme, but must obtain it from extraneous sources, such as the host, the food supply, or perhaps from endosymbionts.

## Challenges for the Future

The filarial and schistosome genome sequences now available provide the vanguard for assembly of a genome sequence catalog of the numerous other neglected helminth parasites (Table 1). Comparative genomics will likely be a dominant approach to organize, interpret, and utilize the vast amounts of genomic information anticipated from the genomes of these parasites (e.g. [83],[84]). In terms of sequencing tools, the new generation of “massively parallel” sequencing platforms commercially available today, (such as the Roche/454 pyrosequencer [85], Illumina/Solexa [86], and SOLiD [87]) offer of the order of 100- to 1,000-fold increases in throughput over the Sanger sequencing technology [88] on capillary electrophoresis instruments. This rapid change to producing millions of DNA sequence reads in a short time will have a huge impact on research on NTDs. Each platform has a specific application: while the Roche/454 is optimal for in-depth analysis of whole transcriptomes and de novo sequencing of bacterial and small eukaryotic genomes, the Illumina and SOLiD systems are highly attractive for resequencing projects aimed at identifying genetic variants (mutations, insertions, deletions), profiling and discovering noncoding RNAs (ncRNAs), and studying epigenetic modifications of histones and DNA. With the increased read length and improved error rate of massively parallel pyrosequencing technology, de novo sequencing of helminth genomes has become possible at a fraction of earlier costs. In the next five years, projects at the Washington University's Genome Center (http://www.genome.gov/10002154) and the Wellcome Trust Sanger Institute (http://www.sanger.ac.uk/Projects/Helminths/) should increase the available sequence data on human helminths and their close relatives by an order of magnitude, adding more than 20 draft genomes to those listed in Table 1.

Once these reference genomes become available, sequencing of clinical isolates is expected to accelerate. Sequencing of the clinical strains and strain-to-reference comparisons can be performed using platforms such as Illumina/Solexa and SOLiD to investigate genome-wide polymorphism and provide a comprehensive picture of natural helminth genome variation. These approaches should also be valuable for exploring genetic changes involved in resistance to anti-worm drugs and understanding the potential mechanisms of drug resistance in human parasites, and can be expected to facilitate development of genetic markers to monitor and manage any future appearance and spread of drug resistance. These phenomena are of tremendous importance, particularly since some major neglected helminth diseases are being targeted in mass drug treatment campaigns [89]. In addition, the new generation of sequencing technologies has also provided unprecedented opportunities for high-throughput functional genomic research (reviewed in [90]) that awaits application to helminth research.

Although some details of immunomodulation by helminth components have been characterized, we are just beginning to understand how these parasite products act on immune responses and to assemble fragmentary information on individual components into a comprehensive picture. Comparisons of helminth molecules with orthologues/paralogues from free-living relatives will strengthen efforts to decipher the strategies adopted by helminth parasites to evade and subvert their host immune responses. This information will be exploitable for development of drugs and vaccines against the parasites and potentially also novel therapeutic biologics for use in humans. Future studies might determine whether helminth proteins with unknown function might be the source for the intriguing regulatory effects helminth infections have on the host immune response.

Treatment for helminthic infections, responsible for hundreds of thousands of deaths each year, depends almost exclusively on just two or three drugs: praziquantel, the benzimidazoles, and ivermectin. Vaccines and new drugs are needed, certainly because drug resistance in human helminth parasites such as schistosomes, whipworms, and filariae, to these compounds would present a major problem for current treatment and control strategies. Pharmacogenomics with the new helminth genomes represents a practicable route forward toward new drugs. For example, chemogenomics screening of the genome sequence of *S. mansoni* identified >20 parasite proteins for which potential drugs are available approved for other human ailments [25], and indeed for which, in the case of the schistosome thioredoxin glutathione reductase, auranofin (an anti-arthritis medication) was shown recently to exhibit potent anti-schistosomal activity [91]. Finally, the vast new sequence information will undoubtedly allow revision of our understanding of the host–parasite relationship, its evolution, vector–pathogen and helminth–symbiont interactions, unique aspects of cell biology and biochemistry, phylogenetic relationships, intervention targets, research approaches (e.g. [92]), and so forth.

## References

[1] Hotez PJ, Brindley PJ, Bethony JM, King CH, Pearce EJ (2008). Helminth infections: The great neglected tropical diseases.. J Clin Invest.

[2] Hotez PJ, Kamath A (2009). Neglected tropical diseases in sub-Saharan Africa: Review of their prevalence, distribution, and disease burden.. PLoS Negl Trop Dis.

[3] Patz JA, Graczyk TK, Geller N, Vittor AY (2000). Effects of environmental change on emerging parasitic diseases.. Int J Parasitol.

[4] Liang S, Yang C, Zhong B, Qiu D (2006). Re-emerging schistosomiasis in hilly and mountainous areas of Sichuan, China.. Bull WHO.

[5] Huyse T, Webster BL, Geldof S, Stothard JR, Diaw OT (2009). Bidirectional introgressive hybridization between a cattle and human schistosome species.. PLoS Pathog.

[6] Kalinna BH, Brindley PJ (2007). Manipulating the manipulators: Advances in parasitic helminth transgenesis.. Trends Parasitol.

[7] Krasky A, Rohwer A, Schroeder J, Selzer PM (2007). A combined bioinformatics and chemoinformatics approach for the development of new antiparasitic drugs.. Genomics.

[8] Mitreva M, Zarlenga DS, McCarter JP, Jasmer DP (2007). Parasitic nematodes - From genomes to control.. Vet Parasitol.

[9] Berriman M, Lustigman S, McCarter JP (2007). Helminth initiative for drug discovery – Report of the informal consultation, genomics and emerging drug discovery technologies.. Expert Opin Drug Discovery.

[10] Lustigman S, Ford S, Crawford MJ, Lyland RogerT, Browning IrvingB (2008). RNA Interference: from functional genomics to validation of drug targets in helminths.. RNA interference research progress.

[11] Franco GR, Adams MD, Soares MB, Simpson AJG, Venter JC (1995). Identification of new *Schistosoma mansoni* genes by the EST strategy using a directional cDNA library.. Gene.

[12] Gobert GN, Moertel L, Brindley PJ, McManus DP (2009). Developmental gene expression profiles of the human pathogen *Schistosoma japonicum*.. BMC Genomics.

[13] Robinson MW, Connolly B (2005). Proteomic analysis of the excretory-secretory proteins of the Trichinella spiralis L1 larva, a nematode parasite of skeletal muscle.. Proteomics.

[14] Mitreva M, McCarter JP, Martin J, Dante M, Wylie T (2004). Comparative genomics of gene expression in the parasitic and free-living nematodes *Strongyloides stercoralis* and *Caenorhabditis elegans*.. Genome Res.

[15] Taft AS, Vermeire JJ, Bernier J, Birkeland SR, Cipriano MJ (2009). Transcriptome analysis of Schistosoma mansoni larval development using serial analysis of gene expression (SAGE).. Parasitology.

[16] Mitreva M, McCarter JP, Arasu P, Hawdon J, Martin J (2005). Investigating hookworm genomes by comparative analysis of two *Ancylostoma* species.. BMC Genomics.

[17] McCarter JP (2004). Genomic filtering: An approach to discovering novel antiparasitics.. Trends Parasitol.

[18] Wasmuth J, Schmid R, Hedley A, Blaxter M (2008). On the extent and origins of genic novelty in the phylum Nematoda.. PloS Negl Trop Dis.

[19] Yin Y, Martin J, Abubucker S, Wang Z, Wyrwicz L (2009). Molecular determinants archetypical to the phylum Nematoda.. BMC Genomics.

[20] Wang Z, Martin J, Abubucker S, Yin Y, Gasser R (2009). Systematic analysis of insertions and deletions specific to nematode proteins and their proposed functional and evolutionary relevance.. BMC Evol Biol.

[21] The *C. elegans* Sequencing Consortium (1998). Genome sequence of the nematode *C. elegans*: A platform for investigating biology.. Science.

[22] Ghedin E, Wang S, Spiro D, Caler E, Zhao Q (2007). Draft genome of the filarial nematode parasite *Brugia malayi*.. Science.

[23] Abubucker S, Martin J, Yin Y, Fulton L, Yang S-P (2008). The canine hookworm genome: Analysis and classification of *Ancylostoma caninum* survey sequences.. Mol Biochem Parasitol.

[24] Liu F, Zhou Y, Wang ZQ, Lu G, *Schistosoma japonicum* Genome Sequencing and Functional Analysis Consortium (2009). The *Schistosoma japonicum* genome reveals features of host-parasite interplay.. Nature.

[25] Berriman M, Haas BJ, LoVerde PT, Wilson RA, Dillon GP (2009). The genome of the blood fluke *Schistosoma mansoni*.. Nature.

[26] Dunn CW, Hejnol A, Matus DQ, Pang K, Browne WE (2008). Broad phylogenomic sampling improves resolution of the animal tree of life.. Nature.

[27] Krautz-Peterson G, Bhardwaj R, Faghiri Z, Tararam C, Skelly PJ (2009). RNA interference in schistosomes: machinery and methodology.. Parasitology.

[28] Mann VH, Morales ME, Kines KJ, Brindley PJ (2008). Transgenesis of schistosomes: approaches using mobile genetic elements.. Parasitology.

[29] Freitas TC, Jung E, Pearce EJ (2007). TGF-beta signaling controls embryo development in the parasitic flatworm *Schistosoma mansoni*.. PLoS Pathog.

[30] Morales ME, Rinaldi G, Kines KJ, Gobert GN, Tort JF (2008). RNA interference targeting *Schistosoma mansoni* cathepsin D, the apical enzyme of the hemoglobin proteolysis cascade.. Mol Biochem Parasitol.

[31] Rinaldi G, Morales ME, Alrefaei YN, Cancela M, Castillo E (2009). RNA interference targeting leucine aminopeptidases inhibits hatching of eggs of the human blood fluke, *Schistosoma mansoni*.. Mol Biochem Parasitol.

[32] Faghiri Z, Skelly PJ (2009). The role of tegumental aquaporin from the human parasitic worm, *Schistosoma mansoni*, in osmoregulation and drug uptake.. FASEB J.

[33] Ford L, Zhang J, Liu J, Hashmi S, Fuhrman JA (2009). Functional analysis of the cathepsin-like cysteine protease genes in adult *Brugia malayi* using RNA interference.. PLoS Negl Trop Dis.

[34] Morales ME, Mann VH, Kines KJ, Gobert GN, Kalinna BH (2007). *piggyBac* transposon mediated transgenesis of the human blood fluke, *Schistosoma mansoni*.. FASEB J.

[35] Kines KJ, Mann VH, Morales ME, Shelby BD, Kalinna BH (2006). Transduction of *Schistosoma mansoni* by vesicular stomatitis virus glycoprotein-pseudotyped Moloney murine leukemia retrovirus.. Exp Parasitol.

[36] Kines KJ, Morales ME, Mann VH, Gobert GN, Brindley PJ (2008). Integration of reporter transgenes into *Schistosoma mansoni* chromosomes mediated by pseudotyped murine leukemia virus.. FASEB J.

[37] Li X, Massey HC, , Nolan TJ, Schad GA, Kraus K (2006). Successful transgenesis of the parasitic nematode *Strongyloides stercoralis* requires endogenous non-coding control elements.. Int J Parasitol.

[38] Castelletto ML, Massey HC, , Lok JB (2009). Morphogenesis of *Strongyloides stercoralis* infective larvae requires the DAF-16 ortholog FKTF-1.. PLoS Pathog.

[39] de Oliveira A, Katholi CR, Unnasch TR (2008). Characterization of the promoter of the *Brugia malayi* 12 kDa small subunit ribosomal protein (RPS12) gene.. Int J Parasitol.

[40] Rogers A, Antoshechkin I, Bieri T, Blasiar D, Bastiani C (2008). Wormbase 2007.. Nucleic Acids Res 36(Database issue).

[41] Mitreva N, Appleton J, McCarter JP, Jasmer DP (2005). Expressed sequence tags from life cycle stages of *Trichinella spiralis*: Application to biology and parasite control.. Vet Parasitol.

[42] Martin J, Abubucker S, Wylie T, Yin Y, Mitreva M (2009). Nematode.net update 2008: Improvements enabling more efficient data mining and comparative nematode genomics.. Nucleic Acids Res.

[43] Parkinson J, Whitton C, Schmid R, Thomson M, Blaxter M (2004). NEMBASE: A resource for parasitic nematode ESTs.. Nucleic Acids Res.

[44] Zerlotini A, Heiges M, Wang H, Moraes RL, Dominitini AJ (2009). SchistoDB: A *Schistosoma mansoni* genome resource.. Nucleic Acids Res.

[45] Liu F, Chen P, Cui SJ, Wang ZQ, Han ZG (2008). SjTPdb: Integrated transcriptome and proteome database and analysis platform for *Schistosoma japonicum*.. BMC Genomics.

[46] Robb SMC, Ross E, Sánchez Alvarado A (2008). SmedGD: The *Schmidtea mediterranea* genome database.. Nucleic Acids Res 36(Database issue).

[47] Blaxter ML, De Ley P, Garey JR, Liu LX, Scheldeman P (1998). A molecular evolutionary framework for the phylum Nematoda.. Nature.

[48] Holterman M, van der Wurff A, van den Elsen S, van Megen H, Bongers T (2006). Phylum-wide analysis of SSU rDNA reveals deep phylogenetic relationships among nematodes and accelerated evolution toward crown clades.. Mol Biol Evol.

[49] Mitreva M, Smant G, Helder J (2009). Role of horizontal gene transfer in the evolution of plant parasitism among nematodes. In: Horizontal Gene Transfer.. Methods Mol Biol.

[50] Abad P, Gouzy J, Aury J-M, Castagnone-Sereno P, Danchin EG (2008). Genome sequence of the metazoan plant-parasitic nematode *Meloidogyne incognita*.. Nat Biotech.

[51] Opperman CH, Bird DM, Williamson VM, Rokhsar DS, Burke M (2008). Sequence and genetic map of *Meloidogyne hapla*: A compact nematode genome for plant parasitism.. Proc Natl Acad Sci U S A.

[52] Dieterich C, Clifton SW, Schuster LN, Chinwalla A, Delehaunty K (2008). The *Pristionchus pacificus* genom*e* provides a unique perspective on nematode lifestyle and parasitism.. Nat Genet.

[53] Robb SM, Ross E, Sánchez Alvarado A (2008). SmedGD: The *Schmidtea mediterranea* genome database.. Nucleic Acids Res.

[54] Maizels RM, Balic A, Gomez-Escobar N, Nair M, Taylor MD (2004). Helminth parasites–Masters of regulation.. Immunol Rev.

[55] Ohnmacht C, Voehringer D (2009). Basophil effector function and homeostasis during helminth infection.. Blood.

[56] Hartmann S, Kyewski B, Sonnenburg B, Lucius R (1997). A filarial cysteine protease inhibitor down-regulates T cell proliferation and enhances interleukin-10 production.. Eur J Immunol.

[57] Hartmann S, Lucius R (2003). Modulation of host immune responses by nematode cystatins.. Int J Parasitol.

[58] Harnett W, McInnes IB, Harnett MM (2004). ES-62, a filarial nematode-derived immunomodulator with anti-inflammatory potential.. Immunol Lett.

[59] Gomez-Escobar N, Lewis E, Maizels RM (1998). A novel member of the transforming growth factor-beta (TGF-beta) superfamily from the filarial nematodes *Brugia malayi* and *B. pahangi*.. Exp Parasitol.

[60] Gomez-Escobar N, Gregory WF, Maizels RM (2000). Identification of tgh-2, a filarial nematode homolog of *Caenorhabditis elegans* daf-7 and human transforming growth factor beta, expressed in microfilarial and adult stages of *Brugia malayi*.. Infect Immun.

[61] van der Kleij D, Latz E, Brouwers JF, Kruize JC, Schmitz M (2002). A novel host-parasite lipid cross-talk. Schistosomal lyso-phosphatidylserine activates toll-like receptor 2 and affects immune polarization.. J Biol Chem.

[62] Pastrana DV, Raghavan N, FitzGerald P, Eisinger SW, Metz C (1998). Filarial nematode parasites secrete a homologue of the human cytokine macrophage migration inhibitory factor.. Infect Immun.

[63] Hewitson JP, Grainger JR, Maizels RM (2009). Helminth immunoregulation: The role of parasite secreted proteins in modulating host immunity.. Mol Biochem Parasitol.

[64] Yazdanbakhsh M, van den Biggelaar A, Maizels RM (2001). Th2 responses without atopy: Immunoregulation in chronic helminth infections and reduced allergic disease.. Trends Immunol.

[65] Imai S, Fujita K (2004). Molecules of parasites as immunomodulatory drugs.. Curr Top Med Chem.

[66] Harnett W, Harnett MM (2008). Therapeutic immunomodulators from nematode parasites.. Expert Rev Mol Med.

[67] Harnett W, Harnett MM (2008). Parasitic nematode modulation of allergic disease.. Curr Allergy Asthma Rep.

[68] Johnston MJ, MacDonald JA, McKay DM (2009). Parasitic helminths: A pharmacopeia of anti-inflammatory molecules.. Parasitology.

[69] McKay DM (2009). The therapeutic helminth?. Trends Parasitol.

[70] Erb KJ (2009). Can helminths or helminth-derived products be used in humans to prevent or treat allergic diseases?. Trends Immunol.

[71] Summers RW, Elliott DE, Urban JF, , Thompson R, Weinstock JV (2005). *Trichuris suis* therapy in Crohn's disease.. Gut.

[72] Summers RW, Elliott DE, Urban JF, , Thompson RA, Weinstock JV (2005). *Trichuris suis* therapy for active ulcerative colitis: A randomized controlled trial.. Gastroenterology.

[73] Lima C, Perini A, Garcia ML, Martins MA, Teixeira MM (2002). Eosinophilic inflammation and airway hyper-responsiveness are profoundly inhibited by a helminth (*Ascaris suum*) extract in a murine model of asthma.. Clin Exp Allergy.

[74] Schnoeller C, Rausch S, Pillai S, Avagyan A, Wittig BM (2008). A helminth immunomodulator reduces allergic and inflammatory responses by induction of IL-10-producing macrophages.. J Immunol.

[75] Melendez AJ, Harnett MM, Pushparaj PN, Wong WS, Tay HK (2007). Inhibition of Fc epsilon RI-mediated mast cell responses by ES-62, a product of parasitic filarial nematodes.. Nat Med.

[76] McInnes IB, Leung BP, Harnett M, Gracie JA, Liew FY (2003). A novel therapeutic approach targeting articular inflammation using the filarial nematode-derived phosphorylcholine-containing glycoprotein ES-62.. J Immunol.

[77] Donnelly S, O'Neill SM, Sekiya M, Mulcahy G, Dalton JP (2005). Thioredoxin peroxidase secreted by *Fasciola hepatica* induces the alternative activation of macrophages.. Infect Immun.

[78] Holland MJ, Harcus YM, Riches PL, Maizels RM (2000). Proteins secreted by the parasitic nematode *Nippostrongylus brasiliensis* act as adjuvants for Th2 responses.. Eur J Immunol.

[79] Han ZG, Brindley PJ, Wang S, Chen Z (2009). Schistosome genomics: New perspectives on schistosome biology and host parasite interaction.. Annu Rev Genomics Hum Genet.

[80] Foster J, Ganatra M, Kamal I, Ware J, Makarova K (2005). The *Wolbachia* genome of *Brugia malayi*: endosymbiont evolution within a human pathogenic nematode.. PLoS Biol.

[81] Park J, Kim KJ, Choi K-S, Grab DJ, Dumler JS (1993). *Anaplasma phagocytophilum* AnkA binds to granulocyte DNA and nuclear proteins.. Cell Microbiol.

[82] Warbrick EV, Barker GC, Rees HH, Howells RE (1993). The effect of invertebrate hormones and potential hormone inhibitors on the third larval moult of the filarial nematode, *Dirofilaria immitis*, in vitro.. Parasitology.

[83] Nisbet AJ, Cottee PA, Gasser RB (2008). Genomics of reproduction in nematodes: prospects for parasite intervention?. Trends Parasitol.

[84] Dieterich C, Sommer RJ (2009). How to become a parasite - Lessons from the genomes of nematodes.. Trends Genet.

[85] Margulies M, Egholm M, Altman WE, Attiya S, Bader JS (2005). Genome sequencing in microfabricated high-density picolitre reactors.. Nature.

[86] Bennett S (2004). Solexa Ltd.. Pharmacogenomics.

[87] Shendure J, Porreca GJ, Reppas NB, Lin X, McCutcheon JP (2005). Accurate multiplex polony sequencing of an evolved bacterial genome.. Science.

[88] Sanger F, Niklen S, Coulson A (1977). DNA sequencing with chain-terminating inhibitors.. Proc Natl Acad Sci U S A.

[89] Fenwick A (2009). Host-parasite relations and implications for control.. Adv Parasitol.

[90] Morozova O, Marra MA (2008). Applications of next-generation sequencing technologies in functional genomics.. Genomics.

[91] Kuntz AN, Davioud-Charvet E, Sayed AA, Califf LL, Dessolin J (2007). Thioredoxin glutathione reductase from *Schistosoma mansoni*: An essential parasite enzyme and a key drug target.. PLoS Med.

[92] Cosseau C, Azzi AH, Smith K, Freitag M, Mitta G (2009). Native chromatin immunoprecipitation (N-ChIP) and ChIP-Seq of *Schistosoma mansoni*: Critical experimental parameters.. Mol Biochem Parasitol.

[93] Chenna R, Sugawara H, Koike T, Lopez R, Gibson TJ (2003). Multiple sequence alignment with the Clustal series of programs.. Nucleic Acids Res.

[94] Felsenstein J (1988). Phylogenies from molecular sequences: Inference and reliability.. Ann Rev Genet.

